# Common mental disorders among patients attending monk healers and primary health care centres in Thailand: a cross-sectional study

**DOI:** 10.1186/s13033-020-00414-2

**Published:** 2020-11-04

**Authors:** Supa Pengpid, Karl Peltzer

**Affiliations:** 1 ASEAN Institute for Health Development, Mahidol University, Salaya, Phutthamonthon, Nakhon Pathom Thailand; 2 Department of Research Administration and Development, University of Limpopo, Turfloop, South Africa; 3 Department of Psychology, University of the Free State, Bloemfontein, South Africa

**Keywords:** Common mental disorders, Monk healer, Primary care, Thailand

## Abstract

**Background:**

This study aimed to assess the rate of common mental disorders in patients consulting monk healers or health centres in Thailand.

**Methods:**

Patients consecutively consulting monk healers or health centres were assessed with screening measures of three common mental disorders (major depressive, general anxiety and somatization disorder).

**Results:**

The prevalence of any common mental disorder was significantly higher in patients attending monk healers (31.1%) than those attending primary care health centres (22.3%) (P < 0.001). Likewise, the prevalence of each common mental disorder was significantly higher in clients attending monk healers (major depressive disorder 21.0%, generalized anxiety disorder 8.1%, and somatization disorder 19.0%) than in patients attending health centres (major depressive disorder 15.8%, generalized anxiety disorder 3.5%, and somatization disorder 12.5%). In adjusted logistic regression analysis among patients of monk healers, female sex, being single, divorced, separated or widowed, and low social support were associated with any common mental disorder. Among patients of a health centre, lower education, not employed, high debt status and low social support were associated with any common mental disorder.

**Conclusion:**

The study found a higher prevalence of common mental disorders in patients consulting monk healers than primary care centre attendees, calling for integrated management of common mental disorders.

## Background

Traditional and faith healers have been identified as a workforce contributing to mental health care worldwide [1]. In a systematic review, traditional healers have shown to be beneficial in psychosocial interventions by reducing psychological distress and mild symptoms in common mental disorders (= CMD) [1]. Among non-communicable disease and mental disorder patients in Thailand, 26.3% had been utilizing traditional and/or faith healing practitioners in the past 12 months [2]. Traditional health practitioners that may include spiritual, monk, and herbal healers, are located in all different parts of Thailand [3]. Monk healers (*maw pra*) that reside at Buddhist temples provide various types of treatments to patients, including prayers and Thai traditional medicine [4, 5]. Several studies [6, 7] have described the management of CMD by monk healers in Thailand [6, 7].

Studies investigating the prevalence of CMD in the traditional health practitioner setting and comparative studies in the primary health care system are scarce, in particular in Southeast Asia. Several studies on the prevalence of CMD in the traditional health practitioner setting have been conducted in Africa, e.g. in Kenya the prevalence of depression was 22.9% [8], in Tanzania, the prevalence of CMD was 48% (double that of primary care patients 24%) [9], and in Uganda the prevalence of psychological distress was 65.1% [10]. A number of studies investigated the prevalence of CMD in a primary care setting in Asia, e.g., in Thailand, the prevalence of depression (mild to severe) was 11.5% [11], in Nepal, the prevalence of depression was 16.8% [12], in Kuwait the prevalence of CMD was 42.7% [13], in Singapore, the prevalence of major/minor depressive disorders was 9% [14], and in China, the prevalence of moderate or high somatic symptoms (PHQ-15: scores ≥ 10) was 19.0%, depression 15.2%, anxiety 6.9%, both depression and anxiety 5.2% [13].

Determinants of CMD of persons attending traditional health practitioners, include being older [8, 9], being female [8], single, divorced or separated [8, 9], Christian [9], lack of education [8], better educated [9], unemployed [8], lack of food and being in debt [10]. Determinants of CMD of persons attending primary health facilities include lack of social support and life stress [11]. This study aimed to assess the rate of CMD in patients presenting to monk healers or primary care health facilities in Thailand.

## Methods

### Participants and procedures

In a cross-sectional study design, adult patients attending primary care health centres or monk healers were systematically recruited (consecutive sampling) after written informed consent was obtained. Purposeful sampling was used to select three monk healers or temples and three primary health care centres located in four districts of the eastern and central region of Thailand. Inclusion criteria for the selection of the study sites were to have at least five patients a day, and the inclusion criteria for the selection of clients or patients was aged 18 years and above. The study was conducted from November 2018 to February 2019. A professional nurse conducted face-to-face interviews in Thai language with patients on background data and CMD. Questionnaires were pretested for validity on a sample of 30 patients, not included in the final sample. Research nurses were systematically trained in the administration of the questionnaires. Moreover, the assessment procedures and implementation were routinely monitored by senior research staff. Study approval was obtained from each of the study sites, and the study protocol was approved by the “Office of The Committee for Research Ethics (Social Sciences), Mahidol University (No.: 2017/055.1403).”

### Measures

*Sociodemographic data* included marital status, education, sex, age, work status, religion, and economic status (extent of debt).

Social support was assessed with the “Oslo 3-items Social Support Scale (OSSS-3)”, covering “the number of people the respondent feels close to, the interest and concern shown by others, and the ease of obtaining practical help from other.” [16]. The total scores (3–14) were grouped into “3–8 = poor, 9–11, = moderate, and 12–16 strong support” [14] (Cronbach’s alpha 0.75 in this sample).

The *Patient Health Questionnaire-9* (PHQ-9) was used to assess major depressive disorder (MDD)” [17]. “It has demonstrated high sensitivity (0.84) and specificity (0.77) in a validation study in Thailand, using a cut-off score of nine or more as indicative for MDD” [18] (Cronbach’s alpha 0.88 in this sample).

The *Generalized anxiety disorder* 7-item (GAD-7) scale was used to measure “the severity of generalized anxiety, with a score of 10 or more indicating moderate or severe GAD” [19] (Cronbach’s alpha 0.92 in this sample).

The *Patient Health Questionnaire-15 somatic symptoms (PHQ-15)* screened for somatization disorder [20]. “The somatic symptoms severity is calculated by assigning scores at 0, 1, and 2 to the response categories of not at all, bothered a little, and bothered a lot for the 15 somatic symptoms” [20]. As recommended in previous research [20–22], cut-off scores ≥ 10 indicated moderate or high somatic symptom severity [20–22] (Cronbach’s alpha 0.83).

### Data analysis

Descriptive statistics (frequencies, percentages, means, and standard deviations) is used to show the prevalence of CMD and sample characteristics. Differences in proportions were tested with Pearson chi-square tests and parametric tests. Univariate and multivariable logistic regression analyses were utilized to estimate demographic and social determinants of CMD by health care settings. The data were analysed with IBM-SPSS for Windows, version 25 (Chicago, IL, USA).

## Results

### Sample and common mental disorder characteristics

The total sample consisted of 1251 patients, 51.5% from health centres and 48.5% from monk healers; the overall response rate was 97%. The proportion of female patients with the monk healers was 76.6% and with the health facility sites 72.5%, respectively), and all patients were Buddhists by religion. Patients attending monk healers had higher socioeconomic status (education, less debt), social support and were younger than primary care attendees. The prevalence of any CMD was significantly higher in clients attending monk healers (31.1%) than those attending primary care health centres (22.3%) (P < 0.001) (see Table 1).

**Table 1:** Sociodemographic and any common mental disorder characteristics of participants attending monk healers and primary care health centres (N = 1251)

Variable	Monk healer	Health centre	P value	Monk healer	Health centre	P value
	Sample	Sample		CMD	CMD	
	M (SD)	M (SD)		M (SD)	M (SD)	
Age in years	47.3 (13.8)	53.3 (14.1)	< 0.001	45.1 (13.5)	56.2 (14.4)	< 0.001
	N (%)	N (%)		N (%)	N (%)	
All	607 (48.5)	644 (51.5)		181 (31.1)	138 (22.3)	
Sex						
Female	465 (76.6)	469 (72.8)	0.254	152 (84.0)	99 (71.7)	0.007
Male	142 (23.4)	175 (27.2)		29 (16.0)	39 (28.3)	
Formal education						
Primary or less	225 (38.5)	404 (64.6)	< 0.001	57 (32.6)	110 (80.3)	< 0.001
Secondary	185 (31.7)	166 (26.6)		63 (36.0)	21 (15.3)	
Post-secondary	174 (29.8)	55 (8.8)		55 (31.4)	6 (4.4)	
Marital status						
Single/divorced/separated/widowed	250 (41.7)	145 (22.9)	< 0.001	97 (54.2)	31 (22.8)	< 0.001
Married	350 (58.3)	489 (77.1)		82 (45.8)	105 (77.2)	
Employment status						
No	192 (32.0)	169 (26.4)	0.127	69 (39.2)	59 (43.4)	0.457
Yes	408 (68.0)	467 (73.6)		107 (60.8)	77 (56.6)	
In debt						
No/little	436 (71.8)	474 (73.6)	0.481	124 (68.5)	57 (31.5)	0.002
High	171 (28.2)	170 (26.4)		57 (31.5)	67 (48.6)	
Social support						
Low	106 (17.7)	106 (16.7)	< 0.001	49 (27.1)	49 (35.8)	0.201
Medium	280 (46.7)	395 (62.3)		75 (41.4)	54 (39.4)	
High	213 (35.6)	133 (21.0)		57 (31.5)	34 (24.8)	

*M *mean, *SD *standard deviation, *CMD *common mental disorder

The prevalence of each CMD was significantly higher in clients attending monk healers (major depressive disorder 21.0%, generalized anxiety disorder 8.1%, and somatization disorder 19.0%) than in patients attending health centres (major depressive disorder 15.8%, generalized anxiety disorder 3.5%, and somatization disorder 12.5%). In addition, the prevalence of the combinations of having two or three different CMD was higher in clients attending monk healers than in patients attending health centres (see Table 2).

**Table 2:** Distribution of the type of common mental disorder by service facility

Type of common mental disorder	Monk healer N (%)	Health centre N (%)	P value
Depressive disorder	123 (21.0)	99 (15.8)	0.021
Anxiety disorder	48 (8.1)	22 (3.5)	< 0.001
Somatization disorder	113 (19.0)	79 (12.5)	0.002
Depressive and anxiety disorder	43 (7.4)	19 (3.0)	< 0.001
Depressive and somatization disorder	60 (10.3)	42 (6.7)	0.027
Anxiety and somatization disorder	26 (4.4)	13 (2.1)	0.021
Depressive, anxiety and somatization disorder	26 (4.5)	13 (2.1)	0.020

### Associations with any common mental disorder

In adjusted logistic regression analysis, among clients of a monk healer, female sex, being single, divorced, separated or widowed, and low social support were associated with any CMD. Among patients of a health centre, lower education, not employed, high debt status, and low social support were associated with any CMD (see Table 3).

**Table 3:** Associations with any common mental disorder by health care setting

Variable	Monk healer		Health centre	
	COR	AOR	COR	AOR
Age in years	0.98 (0.97, 0.99)**	0.98 (0.95, 0.99)*	1.02 (1.01, 1.04)**	1.00 (0.98, 1.01)
Sex				
Female	1 (Reference)	1 (Reference)	1 (Reference)	1 (Reference)
Male	0.27 (0.18, 0.41)***	0.53 (0.32, 0.88)*	0.31 (0.22, 0.44)***	0.88 (0.54, 1.25)
Formal education				
Primary or less	1 (Reference)	1 (Reference)	1 (Reference)	1 (Reference)
Secondary	1.56 (1.01, 2.41)*	1.26 (0.74, 2.14)	0.38 (0.23, 0.63)***	0.37 (0.19, 0.70)**
Post-secondary	1.31 (0.84, 2.03)	0.95 (0.55, 1.64)	0.33 (0.14, 0.79)*	0.24 (0.09, 0.67)**
Marital status				
Single/divorced/separated/widowed	1 (Reference)	1 (Reference)	1 (Reference)	1 (Reference)
Married	0.48 (0.34, 0.69)***	0.60 (0.40, 0.90)*	0.98 (0.62, 1.54)	1.29 (0.74. 2.25)
Employment status				
No	1 (Reference)	1 (Reference)	1 (Reference)	1 (Reference)
Yes	0.63 (0.43, 0.92)*	0.68 (0.44, 1.04)	0.36 (0.24, 0.54)***	0.51 (0.31, 0.85)**
In debt				
No/little	1 (Reference)	1 (Reference)	1 (Reference)	1 (Reference)
High	1.33 (0.91, 1.93)	1.01 (0.66, 1.56)	3.81 (2.58, 5.63)***	2.63 (1.63, 4.26)***
Social support				
Low	1 (Reference)	1 (Reference)	1 (Reference)	1 (Reference)
Medium	0.43 (0.27, 0.67)***	0.54 (0.33, 0.89)*	0.27 (0.17, 0.67)***	0.24 (0.14, 0.42)***
High	0.37 (0.23, 0.60)***	0.52 (0.30, 0.88)*	0.56 (0.33, 0.94)*	0.54 (0.29, 1.03)

*COR* rude odds ratio, *AOR *adjusted odds ratio

***P < 0.001, **P < 0.01, *P < 0.05

## Discussion

The study found a higher prevalence of CMD among monk healers than primary care attenders. Similar results were found in a study in Tanzania [9]. The prevalence of 31.1% of any CMD among monk healer attenders was lower than the one found in Tanzania (48%) [9], and Uganda (psychological distress) (65.1%) [10], and the prevalence of depression (21.0%) among monk healer attenders was similar to a study among traditional health practitioner attenders in Kenya (22.9%) [8]. The prevalence of major depressive disorder was 15.8% among primary care attenders, which was a little higher than in a previous study in a primary care setting in Thailand (11.5%) [11], and Singapore (9%) [14], similar to China (15.2%) [15] and Nepal (16.8%) [12]. The prevalence of CMD among primary care attenders in this study (22.3%) was lower than that found in Kuwait (42.7%) [13], and the prevalence of somatization and generalized anxiety disorder among primary care attenders in this study (12.5% and 3.5%, respectively) was a little lower than found in China (19.0% and 6.9%, respectively) [15].

In agreement with some previous investigations [8, 9], this study found that female sex and being single, divorced, separated, or widowed were associated with the prevalence of any CMD in the monk healer setting. Furthermore, this study found that younger age was associated with CMD in the monk healer setting, while previous studies [8, 9] found that older age was associated with CMD in the traditional health practitioner setting. It is possible that monk healers, rather than traditional Thai health practitioners, similar to the use of complementary medicine, are more attractive to young and middle-class Thais [23]. Some previous studies [8–10] found that educational level, employment status, and economic status were associated with the prevalence of CMD in the traditional health care setting, while this study did not find such differences, only in unadjusted analysis, being unemployed was associated with any CMD in the monk healer setting. Lack of social support was in both care settings (monk and primary care) associated with CMD, which was also found in the primary care setting in Thailand in a previous study [11]. Having lower education, being unemployment, and having high debt were associated with CMD in the primary care setting. This finding seems to show the good accessibility of primary health care centres, also them being free of charge. Furthermore, patients with CMD who are unemployed and have debt should be supported in employment seeking and debt relief.

Considering the high prevalence of CMD in the monk healer setting, it appears that monk healers are better placed to deal with CMD than primary health centres, which are the first population contact for preventive, promotive and basic curative care [24], in Thailand. More research is needed on the treatment approach of monk healers in relation to their diagnosis, management, and treatment outcomes of CMD, such as naturalistic prospective investigations [9]. Monk healers in Thailand could undergo training in evidence-based practices to help in reducing the mental health treatment gap, especially in rural communities [8].

### Study limitations

CMD were only measured using several screening instruments. However, in future studies structured psychiatric interviews, such as with the SCID or SCAN, should be conducted, at least with the ones identified positive with the screening tool. Furthermore, we did not assess the history of health seeking behaviour to show which health system (monk healer, health centre, or other) was utilized serially or parallelly.

## Conclusion

The study found a higher prevalence of CMD among monk healers than primary care attenders in Thailand, calling for integrated management of CMD. Future research should follow up patients presenting to monk healers and primary care health facilities with CMD in Thailand to evaluate treatment outcomes.

## Data Availability

The datasets used and/or analysed during the current study is available from the corresponding author on reasonable request.

## Abbreviations

CMD: Common mental disorders
GAD: Generalized anxiety disorder
MDD: Major depressive disorder
OSSS-3: Oslo 3-items Social Support Scale
PHQ: Patient Health Questionnaire
SCAN: Schedules for Clinical Assessment in Neuropsychiatry
SCID: Structured Clinical Interview for Diagnostic and Statistical Manual for Mental Disorders
